# Parameters of Surface Electromyogram Suggest That Dry Immersion Relieves Motor Symptoms in Patients With Parkinsonism

**DOI:** 10.3389/fnins.2018.00667

**Published:** 2018-09-26

**Authors:** German G. Miroshnichenko, Alexander Yu Meigal, Irina V. Saenko, Liudmila I. Gerasimova-Meigal, Liudmila A. Chernikova, Natalia S. Subbotina, Saara M. Rissanen, Pasi A. Karjalainen

**Affiliations:** ^1^Biosignal Analysis and Medical Imaging Group, Department of Applied Physics, Faculty of Science and Forestry, University of Eastern Finland, Kuopio, Finland; ^2^Laboratory for Novel Methods in Physiology, Institute of High-Tech Biomedical Solutions, Petrozavodsk State University, Petrozavodsk, Russia; ^3^Laboratory of Gravitational Physiology of Sensorimotor System, Department of Sensorimotor Physiology and Countermeasure, Institute of BioMedical Problems, Russian Academy of Sciences, Moscow, Russia; ^4^Department of Human and Animal Physiology, Physiopathology, Histology, Petrozavodsk State University, Petrozavodsk, Russia; ^5^Department of Neurorehabilitation and Physiotherapy, Research Center of Neurology, Russian Academy of Medical Sciences, Moscow, Russia; ^6^Department of Neurology, Psychiatry, and Microbiology, Petrozavodsk State University, Petrozavodsk, Russia

**Keywords:** parkinsonism, dry immersion, microgravity, rehabilitation, electromyogram, nonlinear dynamics, auto-mutual information, determinism

## Abstract

Dry immersion (DI) is acknowledged as a reliable space flight analog condition. At DI, subject is immersed in water being wrapped in a waterproof film to imitate microgravity (μG). Microgravity is known to decrease muscle tone due to deprivation of the sensory stimuli that activate the reflexes that keep up the muscle tone. In contrary, parkinsonian patients are characterized by elevated muscle tone, or rigidity, along with rest tremor and akinesia. We hypothesized that DI can diminish the elevated muscle tone and/or the tremor in parkinsonian patients. Fourteen patients with Parkinson's disease (PD, 10 males, 4 females, 47–73 years) and 5 patients with vascular parkinsonism (VP, 1 male, 4 females, 65–72 years) participated in the study. To evaluate the effect of DI on muscles' functioning, we compared parameters of surface electromyogram (sEMG) measured before and after a single 45-min long immersion session. The sEMG recordings were made from the biceps brachii muscle, bilaterally. Each recording was repeated with the following loading conditions: with arms hanging freely down, and with 0, 1, and 2 kg loading on each hand with elbows flexed to 90°. The sEMG parameters comprised of amplitude, median frequency, time of decay of mutual information, sample entropy, correlation dimension, recurrence rate, and determinism of sEMG. These parameters have earlier been proved to be sensitive to PD severity. We used the Wilcoxon test to decide which parameters were statistically significantly different before and after the dry immersion. Accepting the *p* < 0.05 significance level, amplitude, time of decay of mutual information, recurrence rate, and determinism tended to decrease, while median frequency and sample entropy of sEMG tended to increase after the DI. The most statistically significant change was for the determinism of sEMG from the left biceps with 1 kg loading, which decreased for 84% of the patients. The results suggest that DI can promptly relieve motor symptoms of parkinsonism. We conclude that DI has strong potential as a rehabilitation method for parkinsonian patients.

## 1. Introduction

Parkinson's disease (PD) is acknowledged as one of the major neurological problems due to its epidemiology (de Lau and Breteler, 2006), numerous motor and non-motor symptoms, and functional disability (Alves et al., 2005) in PD patients. From the patients' viewpoint, PD causes dramatic decrease in their safety, wellbeing, and quality of life (Boersma et al., 2016; Fan et al., 2016). From the society's viewpoint, PD patients have increased need for healthcare, which imposes notable economical burden on them, their families, and state (Noyes et al., 2006). PD is characterized by a classic triad of motor symptoms, which include rest tremor, elevated muscle tone, or rigidity, and akinesia. Motor symptoms worsen over time but respond to dopamine replenishment therapy (The Parkinson Study Group, 2004), deep brain stimulation (Vaillancourt et al., 2004), and transcranial magnetic stimulation (Zhu et al., 2015). Still, despite these medical therapies, PD patients develop progressive disability (Alves et al., 2005).

Varied physical interventions have been tried in an effort to find efficient, easy-to-perform, and no costly methods for rehabilitation of PD patients (Tomlinson et al., 2014). The techniques already tested include, e.g., resistance training (Scandalis et al., 2001), robotic-assisted therapy (Picelli et al., 2014), training by means of virtual reality (Yen et al., 2011), whole-body vibration (Ebersbach et al., 2008), and dancing (de Dreu et al., 2015). Also, such exotic therapies as Yoga (Ni et al., 2016), Tai Chi (Zhou et al., 2015), and music therapy (Bukowska et al., 2016) are reported to exert a positive effect on some symptoms of PD. Still, there is a strong need in a rehabilitation technique with a more clear link to pathophysiological mechanisms of parkinsonism. We assume that an unloading technique could be a relevant candidate. Indeed, various unloading techniques, such as weight support (Miyai et al., 2000) and water-related techniques, e.g., aquatic physical therapy (Katsura et al., 2010; Vivas et al., 2011; Volpe et al., 2014) have been reported to improve performance in PD patients.

The phenomenon of muscle tone appears as a commonly used, though often misunderstood concept (Needle et al., 2014). Muscle tone is defined as either the resistance of muscle being passively lengthened (Gordon, 1990), “state of readiness” (Davis et al., 2011), or unconscious “low-level steady-state muscle contraction at rest” (Needle et al., 2014). This prompts that muscle tone is comprised of several distinct components: (1) physical inertia of extremity, (2) the non-reflexive (mechanical-elastic) component, (3) peripheral reflexive muscle contraction, and (4) central neural mechanisms (Katz and Rymer, 1989; Ward, 2000; Needle et al., 2014). The non-reflexive component of muscle tone originates from intrinsic tension between molecules and cells and, correspondingly, it can be measured as a set of viscoelastic characteristics of the skeletal muscle (Demangel et al., 2017). The peripheral reflexive component of muscle tone originates from overactive tonic stretch reflex. On the level of CNS muscle tone is triggered and mediated through the reticular formation, brain stem, cerebellum, extrapyramidal pathways, with modifications from basal ganglia, gamma-motoneurones, and, finally, contraction of intrafusal fibers, which stretch spindle sensory organs, thus initiating activity of alpha-motoneurones via the stretch reflex (Needle et al., 2014). This reflexive activity is aimed at maximizing muscle responsiveness under stressful conditions (Davis et al., 2011). Inhibition from the cortical structures is needed to optimize muscle tone (Guyton and Hall, 2011). Due to complex origins, muscle tone has two different connotations in clinical and research practice. In clinics, muscle tone is used as an easy-to-do bedside measure performed with clinical scales, while for research purposes electromyography (EMG) is useful to assess muscle tone (Ward, 2000).

Two distinct types of exaggerated muscle tone—spasticity and rigidity—are known. Spasticity is hypertonicity caused by misbalance of supraspinal inhibitory and excitatory inputs directed to the spinal cord, leading to a state of disinhibition of the stretch reflex on one side of a joint (Dietz and Sinkjaer, 2007; Trompetto et al., 2014). Instead, rigidity is clinically defined as muscle hypertonicity that persists through the entire range of passive movement on both sides of a particular joint. Also, “unwilled” firing of slow-type motor units might be an important factor in the genesis of rigidity, which is seen as excessive EMG at rest (Cantello et al., 1995). Spasticity is very common in patients with the upper motoneurone lesion (stroke), cerebral palsy and multiply sclerosis (Rivelis and Morice, 2018), while rigidity is the characteristic of patients with Parkinson's disease (PD) and has distinctly different neurophysiological mechanisms (Baradaran et al., 2013).

It is well-known from space physiology that muscle tone in healthy subjects (cosmonauts/astronauts) dramatically decreases within even 1 day in real microgravity on orbit (Kozlovskaya et al., 1988). Tremor is also modified under conditions of space flight (Gallasch et al., 1994). Namely, frequency and amplitude of tremor decrease under weightlessness, presumably due to switch of main source of sensory information from load-dependent muscle reception to position-dependent joint reception (Gallasch et al., 1994). Therefore, we suppose that pathologically elevated muscle tone (rigidity) and tremor may be relieved in PD patients with the help of ground-based analog microgravity techniques. There are few microgravity analog environments: (i) dry immersion (DI), (ii) bed rest, (iii) parabolic airplane flight, (iv) free fall machine, (v) weight support. Among these, DI provides the best microgravity analog due to the following physical factors: (i) supportlessness, (ii) physical inactivity, (iii) whole-body hydrostatic compression (Navasiolava et al., 2011; Watenpaugh, 2016; Demangel et al., 2017).

Effects of DI on the viscoelastic component of muscle tone in healthy subjects are usually seen after 3 days staying at DI (Demangel et al., 2017). However, some studies have demonstrated a much faster effect of analog microgravity on muscles. For example, Schneider et al. (2015) have shown that muscle stiffness changes even after a few seconds of parabolic flight at zero G. Also, Cronin et al. (2016) have demonstrated that muscle spasticity has decreased after 5 min of water immersion due to diminished reflexivity. These data promise that even a short-term DI session, which would be more suitable for older people and PD patients, could exert rehabilitation effect on the motor system. Indeed, the program of several DI sessions with the same patient group as here has been recently reported to exert positive effect on some clinical metrics of PD patients, including scores of depressive mood, UPDRS-III (Unified Parkinson's disease rating scale, motor part), and the rigidity subtotal of the UPDRS-III (Meigal et al., 2018). Tremor subtotal of the UPDRS-III has also decreased, though insignificantly. Still, to date, no instrumented measurement has been conducted to evaluate the effect of a single one DI session on muscle function in PD patients.

Surface electromyography (sEMG) is an affordable, noninvasive, and high-throughput way to get information about muscle functioning. sEMG has been extensively used to examine either normal motor functioning or movement disorders in humans (Farina et al., 2014). Such classical parameters of sEMG as amplitude and spectral frequency allow some estimation of motor unit number and synchronization (Sturman et al., 2005). In recent years, recurrence quantification analysis, entropy and fractal analysis of sEMG provided additional insight in the underlying motor strategies. These parameters characterize a signal in terms of regularity, predictability and self-similarity (Riley and van Orden, 2005). Indeed, nonlinear dynamics based parameters of sEMG surpass the traditional spectral frequency metrics in detection of, for example, fatigue (Sung et al., 2008; Boccia et al., 2016). The nonlinear dynamics based parameters have already been applied for diagnostics of parkinsonism with promising results (Rissanen et al., 2008, 2011; Meigal et al., 2009; Ruonala et al., 2018). They also proved sensitive to neuroleptic-induced parkinsonism in patients with schizophrenia (Meigal et al., 2015). Taken together, nonlinear dynamics based parameters allow quantifying motor unit synchronization and estimating number of independent oscillators generating sEMG (Sturman et al., 2005). Thus, among cardinal motor symptoms of PD, tremor is most reliably characterized by sEMG parameters due to their sensitivity to “hidden rhythms” on electromyogram (Meigal et al., 2013; Oung et al., 2015). In contrary, muscle tone and, presumably, rigidity, are better described by the kinematic parameters collected by wearable inertial sensors (Sáchez-Ferro et al., 2016; di Biase et al., 2018) or by viscoelasticity parameters (Schneider et al., 2015). By using sEMG, rigidity can be seen at best as excessive muscle activity at rest condition (Cantello et al., 1995).

Several studies provide information on the characteristics of sEMG in healthy subjects during analog microgravity. For example, under the condition of 1–8 weeks bed rest, such sEMG parameters as amplitude, median frequency, muscle fiber conduction velocity, and entropy substantially decrease (Portero et al., 1996; Mulder et al., 2009; Cescon and Gazzoni, 2010; Buehring et al., 2011; Fu et al., 2016). Usually, bed rest experiments last for several weeks to simulate space flights of varied duration and lead to substantial impairment in muscle performance, structure, and fiber content (Watenpaugh, 2016). In that respect, bed rest, especially in its most perfect form (with 6° head tilt below horizontal) is likely not reliable for rehabilitation purposes. In contrast, the condition of dry immersion induces microgravity-specific modifications in skeletal muscles much faster than does the bed rest condition (Navasiolava et al., 2011; Watenpaugh, 2016; Demangel et al., 2017). Earlier it has been shown that even a 5 min period of water immersion in waterproof trousers can decrease peripheral reflex excitability after returning to dry land in both healthy controls and post-stroke patients (Cronin et al., 2016). That can be considered as a promise of non-pharmaceutical method of decreasing hyperreflexivity following stroke (Cronin et al., 2016). As such, the condition of short-term dry immersion can be regarded as a potentially reliable method to decrease excessive muscle tone also in PD patients, though neurophysiology of muscle rigidity and spasticity is clearly different.

Therefore, we hypothesize that application of a single one short-term session of DI can diminish tremor and muscle rigidity in PD patients seen as decreased amplitude and modified nonlinear dynamics based parameters of sEMG.

## 2. Patients and methods

### 2.1. Patients

The general inclusion criteria for the patients were that they had an earlier diagnosis of either Parkinson's disease (PD) or vascular parkinsonism (VP). The exclusion criteria are listed in Table 1. Twenty-six patients were clinically examined for participation in this study. Seven of the twenty-six patients were excluded because of elevated blood pressure or extrasystoles on their ECG before a pilot immersion. The remaining 19 of the 26 patients underwent the 15 min pilot dry immersion and further participated in the study. Six of the nineteen patients had controlled arterial hypertension (II–III stage). All the 19 patients gave their informed signed consent before the dry immersion.

**Table 1:** Exclusion criteria for the patients.

**Exclusion reason**	**Exclusion criteria**
Masking or modification of parkinsonian symptoms	Other neurological disorder or injury that interferes motor function
	Osteoporosis
	Recent spinal fracture
	Metabolic disease (e.g., hyperthyreosis, diabetes)
Dry immersion absolute	Epilepsy
contraindications	Mental disorders
	Administration of muscle relaxants
	Cerebral palsy
Dry immersion relative	Hypovolemia
contraindications	Myoma of uterus
(these pathologies are	Atrial fibrillation
allowable during dry	Hemorrhage of various etiology
immersion when controlled	Lung diseases in acute stage
properly, but we still	Oncologic problems
excluded patients who	Myocardial infarction
had them)	Blood clotting disorders (e.g., phlebothrombosis or thrombophlebitis)

Of the 19 patients, 14 had PD (10 males, 4 females, 47–73 years) and 5 had VP (1 male, 4 females, 65–72 years). Their anthropological data, medication, and disease characteristics: duration, form, stage of PD according to Hoehn and Yahr Rating Scale, and Unified Parkinson's disease Rating Scale (UPDRS) are presented in Table 2. All PD patients were recruited through the Department of Neurology, Psychiatry, and Microbiology, Petrozavodsk State University (Petrozavodsk, Russian Federation). This study was approved by Medical Ethic Committee of PetrSU and Ministry of health care and social development of Republic of Karelia.

**Table 2:** Information about the patients.

**No**.	**Age**	**Gender**	**Height (cm)**,	**Disease**	**UPDRS/**	**Stage by**	**Disease &**	**Antiparkinsonian**
	**(yr)**		**weight (kg)**	**duration (yr)**	**UPDRS-III**	**H & Y**	**symptoms**	**medication**
1	62	M	180, 69	7	70 / 37	2	PD, T	Piribedil 200 mg Levodopa 250 mg
								Carbidopa 62.5 mg
								Rasagiline 1.56 mg
								Amantadine 100 mg
								Entocapone 200 mg
2	64	F	160, 81	4	68 / 25	2	PD, T	Piribedil 20 mg
								Benserasid 62.5 mg
3	72	F	154, 66	8	98 / 48	2	VP, T	No medication (never administered)
4	68	M	169, 83	9	49 / 33	2.5	VP, T	Levodopa 975 mg
								Amantadine 300 mg
								Piribedil 150 mg
								Benserasid 25 mg
5	71	M	170, 51	8	43 / 28	2	PD, T	Trihexyphenidyl 6 mg
6	68	F	164, 90	3	59 / 42	2.5	VP, T	Levodopa 225 mg Benserasid 25 mg
								Carbidopa 12.5 mg
								Piribedil 75 mg
7	73	M	172, 61	8	85 / 47	3	PD, T	Piribedil 100 mg Rasagiline 1.56 mg
								Levodopa 250 mg
								Carbidopa 25 mg
8	62	F	169, 81	4	41 / 26	2	PD, T	No medication (for 1 year^*^)
9	57	M	178, 63	6	65 / 48	2.5	PD, T	Piribedil 50 mg Amantadine 200 mg
								Levodopa 250 mg
								Carbidopa 25 mg
10	66	F	160, 67	2	21 / 13	2	VP, T	Levodopa 250 mg Carbidopa 25 mg
								Trihexyphenidyl 6 mg
11	66	F	156, 53	7	82 / 45	2	PD, T	Levodopa 250 mg Carbidopa 25 mg
12	53	M	171, 81	5	49 / 38	1.5	PD, AR	Piribedil 150 mg Amantadine 200 mg
13	70	M	176, 74	2	79 / 49	3	PD, T	Trihexyphenidyl 4 mg
14	68	M	167, 78	3	60 / 32	2	PD, AR	Levodopa 250 mg
								Carbidopa 25 mg
								Pramipexol 50 mg
15	66	F	172, 66	4	57 / 25	3	PD, AR	Levodopa 375 mg
								Carbidopa 37.5 mg
								Piribedil 50 mg
16	47	M	182, 81	3	45 / 16	3	PD, AR	No medication
								(for 1 month^*^)
17	58	M	170, 60	N/A	75 / 37	3	PD, T	Levodopa 300 mg
								Benserasid 75 mg
								Amantadine 200 mg
18	69	M	179, 69	4	60 / 32	3	PD, T	Piribedil 100 mg
								Levodopa 250 mg
								Carbidopa 25 mg
19	65	F	152, 78	7	51 / 15	1	VP, AR	Levodopa 500 mg
								Benserasid 100 mg
								Pramipexole 3 mg
								Amantadine 200 mg

PD, Parkinson's disease; VP, vascular parkinsonism; UPDRS, Unified Parkinson's disease Rating Scale; UPDRS-III, UPDRS motor part III; H & Y, Hoehn and Yahr Rating Scale; T, tremor; AR, akinesia and rigidity;

* *these patients abandoned their therapy themselves*.

### 2.2. Dry immersion procedure

**Figure 1:**
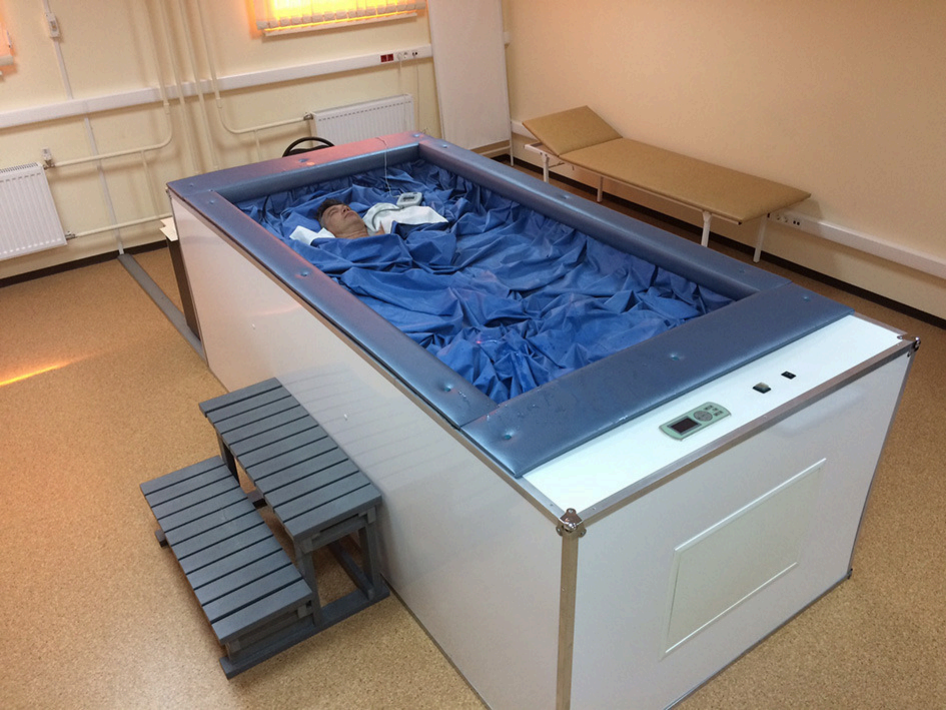
The condition of dry immersion. ^©^ PetrSU/L. Gerasimova-Meigal.

For analog microgravity we utilized the medical system for imitation of weightlessness (MEDSIM, Institute of BioMedical Problems, Moscow, Russia). MEDSIM (Figure 1) appears as a bathtub filled with 2 m^3^ of fresh water. The bathtub is covered by a thin waterproof film of a large size, which allows wrapping subject's body. On the bathtub floor, a motor driven raising platform is mounted. At the initial point the platform is positioned above the water level, enabling the subject to lie down on the film. When a dry immersion procedure starts, the platform lowers down into the water so that the subject stays immersed inside the bathtub wrapped in the film with face and upper part of thorax floating on the water surface. For further details of dry immersion (DI) physics and procedure see Navasiolava et al. (2011).

The patients were instructed to take their medicines at the same time (usually at 7 AM) to standardize conditions of DI and medication. The DI procedure started almost at the same time for each patient (at 9:30 a.m., ±10 min due to differences in time required for blood pressure stabilization). The temperature of water in the DI bathtub was set at 32°C, and it was also filtered and aerated. Before the procedure, the patients visited toilet to urinate, because DI has strong diuretic effect, and drunk a glass of fresh water (200 mL). Then the patients were allowed to adapt to experimental conditions for 10–15 min, lying on the platform wrapped in a cotton sheet to prevent body cooling. By the 10th min blood pressure (UA-767, A&D Company Ltd., Japan) was measured. If blood pressure was not higher than 140/80 mm Hg, the DI procedure was started. The patients were immersed in water in supine position for 45 min, with opportunity to stop the procedure by demand or clinical indications, which were ECG and blood pressure. ECG (Poly-Spectr VNS, Neirosoft Ltd., Ivanovo, Russia) was monitored in lead II to control heart rate and search for extrasystoles. Blood pressure was measured every 15 min (at 15th, 30th, and 45th min).

### 2.3. Measurements of surface electromyogram

The study corresponds to a common pre/post design: we compared surface electromyogram (sEMG) samples before and after a single dry immersion procedure. For our measurements, we used EMG device Neuro-MEP-4 and electrodes from Neurosoft Ltd. (Ivanovo, Russia). We measured sEMG bilaterally from *biceps brachii* muscle. The skin over the muscles was carefully cleaned with a cotton alcohol swab prior to electrodes placement. Abrasive products were not applied to prepare the skin because most of the subjects were older people. Due to the old age, many subjects also had obesity and big skin folds on their arms, which was the reason to choose bipolar plate-mounted electrodes, which have two relevant features. First, they provide closer positioning to the muscle due to their protruding leads. Second, they can be mounted by wrapping adhesive band around the arm, which prevents sliding of the electrode over the muscle. The electrodes were covered with conductive gel (Unimax, Geltek-Medica Company, Moscow, Russia) and applied to the muscle belly longitudinally between the innervation zone and cubital fossa. The electrodes were fixed with adhesive plaster to make sure that sEMG would be recorded from the same sites before and after the immersion. The electrodes were not removed between the pre- and post-immersion measurements. Reference electrode in the form of fabric wrist strap was moistened and attached to the right wrist. Prior to the testing, the subjects were carefully instructed to perform the test correctly, and they were allowed to practice shortly in order to get accustomed with the EMG device and the experimental setting. They were also allowed to warm up their muscles by performing several elbow flexions and extensions. Then we measured sEMG during a submaximal quasi-isometric holding test (Meigal et al., 2012). The recordings were made in standing position in four loading conditions: (1) with arms hanging freely along the trunk, (2) holding elbows flexed to 90° (forearm directed forward and parallel to floor) with palm open and directed upwards (0 kg), (3) with 1 kg, and (4) 2 kg load on each palm. Before each isometric holding, the patient was allowed to rest for a few seconds, after which the next load was introduced. In summary, for each patient we considered 2 body sides and 4 loading conditions and made the measurements before and after the immersion session, which made up at most 16 recordings per patient. Due to technical problems during the measurement, the recording from patient 3: 2 kg after the DI session and the recording from patient 9: 0 kg after the DI session were missing (the patients numbers are from Table 2).

Features and settings of the EMG device and properties of the electrodes were the following. The electrodes (Neurosoft Ltd., Ivanovo, Russia) were made of tin, disk-shaped (8 mm in diameter), with 20 mm spacing between the electrode centers. Input impedance was not less than 200 MΩ, and the measured impedance after electrode placement was not more 10 MΩ. The gain of differential amplifiers was set to 328, the ADC span was ±5 V, the ADC resolution was 16 bits, the peak-to-peak input-referred noise was not more than 5 μV, and the common mode rejection ratio (CMRR) was not less than 100 dB. Each sEMG recording was 3.5 s (70,000 samples) long. The sampling frequency was set to 20 kHz. The 50 Hz notch filter was enabled. The frequency bandwidth was 20–1,000 Hz, and the filters had 3 dB attenuation at the cutoff frequencies. The filters frequency responses corresponded to 1st order high-pass and 2nd order low-pass Butterworth filters.

### 2.4. Preprocessing of surface electromyogram

Data processing and analysis were made using Matlab software (MathWorks Inc., Natick, USA). Preprocessing of the data consisted of removal of power line interference, low-pass filtering, and detrending. Despite of the 50 Hz notch filter in the EMG device, we discovered sharp peaks at multiples of 50 Hz in the spectra of some sEMG recordings, so we applied Fourier interpolation (Mewett et al., 2004) to all the recordings in their frequency domain. The amplitudes in ±1 Hz range around the multiples of 50 Hz were replaced by interpolated values from the line drawn through two points adjacent to the range, while the phases were kept the same. The low-pass filtering was performed using a 14th order elliptic filter designed with Matlab design function (passband frequency 420 Hz, stopband frequency 500 Hz, passband ripple 2·10^−6^ dB, stopband attenuation 80 dB, passband exact match). The filter was applied in both forward and reverse directions to avoid phase distortion. Then the recordings were detrended using the smoothness priors method (Tarvainen et al., 2002) to remove possible movement artifacts. The smoothness priors method is based on regularized least squares and operates as a time-varying FIR high-pass filter with the cutoff frequency gradually changing to zero near the beginning and the end of a signal. Higher values of the only parameter λ correspond to lower cutoff frequencies. We used λ = 10^5^, which corresponds to attenuation of -40 dB at 10 Hz in the middle of a signal sampled at 20 kHz.

### 2.5. Parameters of surface electromyogram

#### 2.5.1. Amplitude and spectral parameters

We calculated amplitude (root mean square) of the recordings and median frequency of power spectrum of the recordings. The power spectrum was calculated as Welch periodogram with segment length of 1 s and 50% segment overlap. The segments spectrum was obtained using fast Fourier transform (Matlab fft function). Hann window was applied to the segments prior to the spectrum calculation to eliminate spectrum distortion because of nonzero values at the segments edges.

#### 2.5.2. Time delay embedding transformation of surface electromyogram

In the nonlinear dynamics based analysis, it is assumed that the examined system is governed by a set of free variables, which follow a trajectory in their space. When the original free variables are unobtainable, the trajectory is replaced by a surrogate obtained from one-dimensional time series. This procedure is called phase space reconstruction and is justified by the embedding theorem (Mañé, 1981; Takens, 1981). As our sEMG recordings were one-dimensional, we applied the time delay embedding transformation to reconstruct the phase space trajectory. The one-dimensional sEMG time series {*x*_1_, *x*_2_, …, *x*_*n*_} was transformed to sequences of vectors in *m*-dimensional space as in Equation (1).

(1) $$\begin{array}{l} X_{i}={\left(x_{i},x_{i+L},x_{i+2L},\ldots ,x_{i+\left(m-1\right)L}\right)}^{T} \end{array}$$

The *L* parameter is called time lag. With properly chosen *m* and *L* parameters, the reconstruction produces a smooth *m*-dimensional trajectory that preserves some features of the presumed original trajectory in a space of free variables of the examined system.

*L* was chosen with the help of mutual information (MI) as advised by Celucci et al. (2003). The MI of a signal and the same signal shifted backwards in time on τ sampling periods was calculated with τ varying from 1 to 500 sampling periods, which produced a dependence MI(τ) for each sEMG recording. To calculate the MI of real-valued signals, we used the algorithm of Fraser and Swinney (1986). We slightly tuned the algorithm: recordings duration was not truncated to the nearest power of two, and the recursive splitting of the histogram bins was stopped when the number of points in a bin was less or equal to a preset value 547, which maximized smoothness of the MI(τ) dependencies. First minimum of MI(τ) (MI decay time) was converted to milliseconds and used as a separate signal parameter, and *L* was chosen as median MI decay time (*L* = 54 sampling periods). We used median instead of mean because the Kolmogorov-Smirnov test showed that the distribution of MI decay time was not normal.

We used false nearest neighbors (FNN) method to choose *m* (Kennel et al., 1992; Celucci et al., 2003). The dependence FNN(*m*) was calculated with *m* varying from 1 to 15, then the embedding dimension was chosen as the point where for all the recordings FNN dropped to 1% of the initial value or lower (*m* = 6).

#### 2.5.3. Correlation dimension and sample entropy

Correlation dimension was chosen as the slope of linear segment of the sEMG correlation integral in double logarithmic scale (Grassberger and Procaccia, 1983). The correlation integral was calculated with Theiler window *W*_CD_ = 270 (*W*_CD_ = (*m* − 1)*L*, Gao and Zheng (1994)). We calculated sample entropy (Richman and Moorman, 2000) using Euclidean interpoint distances and the tolerance distance *r*_SampEn_ = 1.15. The measurements were normalized to unit standard deviation prior to the sample entropy calculation. The tolerance distance *r*_SampEn_ was fitted to maximize the difference between the maximum and minimum values of sample entropy. This fitting, on the one hand, does not imply any assumptions about the effect of dry immersion on sample entropy. On the other hand, it increases the differences of sample entropy between the recordings and, therefore, the potential of sample entropy to distinguish muscles' states.

#### 2.5.4. Recurrence quantification analysis

Recurrence quantification analysis (RQA) parameters were calculated from recurrence plots (Marwan et al., 2007) of the recordings. Recurrence plot (*Rec*) is a tool for visualization of *m*-dimensional phase space trajectories on a plane. *Rec* is a square matrix of ones (black points) and zeros (white points) with a column *i* and a row *j* corresponding to the trajectory points *X*_*i*_ and *X*_*j*_. *Rec*(*i, j*) is equal to one if the corresponding trajectory points *X*_*i*_ and *X*_*j*_ are not further apart from each other than the threshold distance ε and is equal to zero otherwise. Since consecutive (*i* − *j* < *W*) trajectory points tend to be close to each other, their proximity is not informative and is typically not shown on *Rec*, which produces a white strip along the main diagonal of *Rec*. Thus, *Rec* shows the periods of time when the trajectory passes close by its previous locations, which may be movement parallel to an earlier trajectory segment (diagonal lines) or enveloping movement perpendicularly to the segment (vertical lines). RQA quantifies the diagonal and vertical lines (we only quantified the diagonal lines). The recordings were normalized on mean Euclidean interpoint distance ||*X*_*i*_ − *X*_*j*_||, *i* − *j* > 0 prior to the RQA. Then we calculated recurrence rate (share of black points of *Rec*) and determinism (share of black points those form diagonal lines with the length not less than *l*_*min*_). The auxiliary parameters ε, *W*, and minimum diagonal line length *l*_*min*_ were fitted to maximize the span of determinism similarly to sample entropy (ε = 0.7, *W* = 270, *l*_*min*_ = 86). However, in contrary to sample entropy, determinism may take saturated values 0 and 100%, which renders some sEMG recordings indistinguishable by their determinism because they share the same saturated value of it. Therefore, we fitted the auxiliary parameters so that to avoid the saturated values. We also limited the minimum index difference *W* so that *W* ≥ *W*_CD_.

### 2.6. Statistical analysis

The statistical analysis was performed separately for each body side, each loading condition, and each parameter (2 sides × 4 conditions × 7 parameters = 56 statistical tests). The pre-immersion recordings from patients 3 and 4 those did not have the post-immersion counterpart were discarded from the statistical analysis. We used the Wilcoxon test to compare the parameters values before and after the dry immersion. This test quantifies within-subject differences for repeated measures. The reason to use a nonparametric test was that the distributions of the differences were not normal in most of the cases; the normality was tested using the Kolmogorov-Smirnov test. The *p*-values from the Wilcoxon test were compared to the significance level 0.05. We did not apply any correction for multiple comparisons because we had only one research question of whether dry immersion relieves motor symptoms, which are seen by sEMG in parkinsonian patients, so all the comparisons were complementary.

## 3. Results

The general finding was that the post-immersion values of the surface electromyogram (sEMG) parameters tended to be different comparing to the pre-immersion values, excluding the correlation dimension. See the Tables 3, 4 for median values and interquartile ranges of the parameters before and after the dry immersion, and for the percentages of patients for whom the parameters increased or decreased after the dry immersion. In the Tables 3, 4, the 2 pre-immersion recordings with missing post-immersion counterpart are not considered.

**Table 3:** Amplitude (RMS), median frequency, and mutual information (MI) decay time of surface electromyogram before and after the dry immersion (DI).

**Muscle**	**Load**	**Electromyogram**	**Median (IQR)**	**Median (IQR)**	**Drop**	**Rise**	**No change**	***p*-value**
		**parameter**	**before the DI**	**after the DI**	**(%)**	**(%)**	**(%)**	
Right	Arms	Amplitude (μV)	1.7 (1.3, 2.1)	1.2 (0.66, 2.3)	79	21	0	0.024^*^
arm	down	Median frequency (Hz)	73 (36, 107)	90 (74, 111)	42	58	0	0.15
biceps		MI decay time (ms)	4.2 (3.7, 7.2)	3.9 (2.4, 6)	58	37	5	0.21
	0 kg	Amplitude (μV)	15 (8, 25)	12 (8, 19)	67	33	0	0.18
		Median frequency (Hz)	96 (85, 101)	96 (89, 108)	44	56	0	0.25
		MI decay time (ms)	3 (2.6, 3.5)	2.7 (2.5, 3.1)	61	39	0	0.053
	1 kg	Amplitude (μV)	29 (15, 36)	21 (14, 40)	47	53	0	0.57
		Median frequency (Hz)	97 (87, 102)	101 (90, 105)	42	58	0	0.15
		MI decay time (ms)	2.7 (2.6, 3)	2.7 (2.5, 3)	52	37	11	0.32
	2 kg	Amplitude (μV)	34 (19, 56)	36 (21, 49)	50	50	0	0.88
		Median frequency (Hz)	95 (84, 100)	100 (85, 105)	28	72	0	0.053
		MI decay time (ms)	2.7 (2.5, 3.1)	2.6 (2.5, 3.1)	56	33	11	0.13
Left	Arms	Amplitude (μV)	1.6 (0.99, 2.9)	0.91 (0.53, 1.7)	79	21	0	0.016^*^
arm	down	Median frequency (Hz)	74 (27, 123)	114 (34, 175)	32	68	0	0.084
biceps		MI decay time (ms)	4 (2, 11)	2 (1.4, 6.7)	63	26	11	0.062
	0 kg	Amplitude (μV)	14 (8, 21)	11 (4, 19)	78	22	0	0.071
		Median frequency (Hz)	104 (90, 112)	109 (96, 139)	39	61	0	0.064
		MI decay time (ms)	2.5 (2.4, 3)	2.3 (1.8, 3.1)	83	17	0	0.011^*^
	1 kg	Amplitude (μV)	24 (12, 33)	20 (7, 30)	68	32	0	0.024^*^
		Median frequency (Hz)	98 (88, 110)	108 (92, 130)	26	74	0	0.036^*^
		MI decay time (ms)	2.6 (2.3, 3)	2.4 (2, 2.9)	68	21	11	0.055
	2 kg	Amplitude (μV)	30 (16, 48)	24 (9, 36)	78	22	0	0.0029^*^
		Median frequency (Hz)	99 (82, 110)	111 (86, 125)	22	78	0	0.0057^*^
		MI decay time (ms)	2.7 (2.3, 3.3)	2.3 (2, 3)	72	28	0	0.02^*^

*The picture of individual changes of a parameter is described with the percentages of patients for whom the parameter decreased (Drop), increased (Rise), or did not change. The p-values are the results of assessment of a parameter changes using the Wilcoxon test*.

* *p < 0.05; IQR, interquartile range*.

**Table 4:** Nonlinear dynamics based parameters of surface electromyogram before and after the dry immersion (DI).

**Muscle**	**Load**	**Electromyogram**	**Median (IQR)**	**Median (IQR)**	**Drop**	**Rise**	***p*-value**
		**parameter**	**before the DI**	**after the DI**	**(%)**	**(%)**	
Right	Arms	Sample entropy	0.89 (0.73, 0.97)	0.96 (0.84, 1)	16	84	0.0089^*^
arm	down	Correlation dimension	6 (5.6, 6.2)	6 (5.5, 6.1)	53	47	0.81
biceps		Recurrence rate (%)	11 (10, 16)	10 (9, 14)	89	11	0.0089^*^
		Determinism (%)	36 (21, 67)	20 (11, 54)	84	16	0.0079^*^
	0 kg	Sample entropy	0.9 (0.84, 0.92)	0.92 (0.85, 0.95)	33	67	0.18
		Correlation dimension	5.7 (5.2, 6.3)	5.6 (5.2, 5.8)	67	33	0.23
		Recurrence rate (%)	11 (10, 13)	10 (10, 13)	56	44	0.42
		Determinism (%)	49 (44, 60)	46 (37, 62)	56	44	0.1
	1 kg	Sample entropy	0.92 (0.87, 0.95)	0.92 (0.82, 0.94)	53	47	0.97
		Correlation dimension	6 (5.5, 6.4)	6.1 (5.6, 6.6)	42	58	0.57
		Recurrence rate (%)	10 (10, 13)	11 (10, 13)	47	53	0.69
		Determinism (%)	53 (41, 60)	51 (41, 61)	58	42	0.49
	2 kg	Sample entropy	0.93 (0.88, 0.95)	0.94 (0.89, 0.95)	50	50	0.88
		Correlation dimension	6.2 (5.7, 6.5)	5.7 (5, 6.2)	72	28	0.2
		Recurrence rate (%)	10 (9, 12)	10 (9, 12)	44	56	0.4
		Determinism (%)	44 (42, 53)	41 (38, 58)	67	33	0.33
Left	Arms	Sample entropy	0.89 (0.69, 0.97)	0.95 (0.88, 1)	26	74	0.0048^*^
arm	down	Correlation dimension	5.9 (5.6, 6.2)	6 (5.9, 6.3)	37	63	0.44
biceps		Recurrence rate (%)	12 (10, 17)	10 (9, 13)	68	32	0.018^*^
		Determinism (%)	42 (19, 76)	27 (8, 43)	84	16	0.0062^*^
	0 kg	Sample entropy	0.92 (0.71, 0.96)	0.94 (0.81, 0.98)	28	72	0.085
		Correlation dimension	5.8 (5.5, 6.2)	5.9 (5.6, 6.2)	39	61	0.53
		Recurrence rate (%)	11 (10, 15)	11 (10, 15)	50	50	0.78
		Determinism (%)	46 (35, 58)	39 (26, 65)	78	22	0.11
	1 kg	Sample entropy	0.93 (0.85, 0.96)	0.96 (0.92, 0.99)	21	79	0.0033^*^
		Correlation dimension	6 (5.7, 6.2)	5.8 (5.6, 6.4)	53	47	0.69
		Recurrence rate (%)	11 (9, 12)	10 (9, 12)	68	32	0.049^*^
		Determinism (%)	40 (36, 53)	36 (23, 40)	84	16	0.00084^*^
	2 kg	Sample entropy	0.94 (0.91, 0.96)	0.96 (0.93, 0.98)	39	61	0.11
		Correlation dimension	5.9 (5.5, 6.5)	5.9 (5.6, 6.4)	44	56	0.78
		Recurrence rate (%)	10 (9, 12)	10 (9, 11)	44	56	0.81
		Determinism (%)	42 (34, 48)	35 (27, 43)	78	22	0.0065^*^

*The picture of individual changes of a parameter is described with the percentages of patients for whom the parameter decreased (Drop) or increased (Rise). The p-values are the results of assessment of a parameter changes using the Wilcoxon test*.

* *p < 0.05; IQR, interquartile range*.

**Figure 2:**
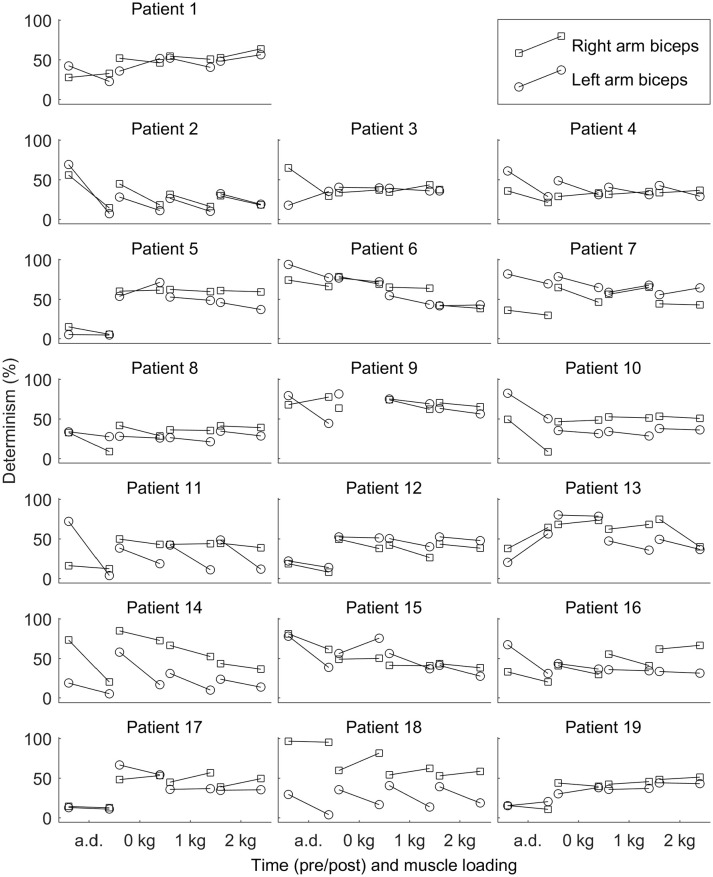
Determinism of surface electromyogram for each patient before and after the dry immersion. The determinism tends to decrease after the dry immersion. Loading conditions are the following: arms down with no load (*a.d*.); 0, 1, and 2 kg loading on each hand with elbow being flexed to 90°.

**Figure 3:**
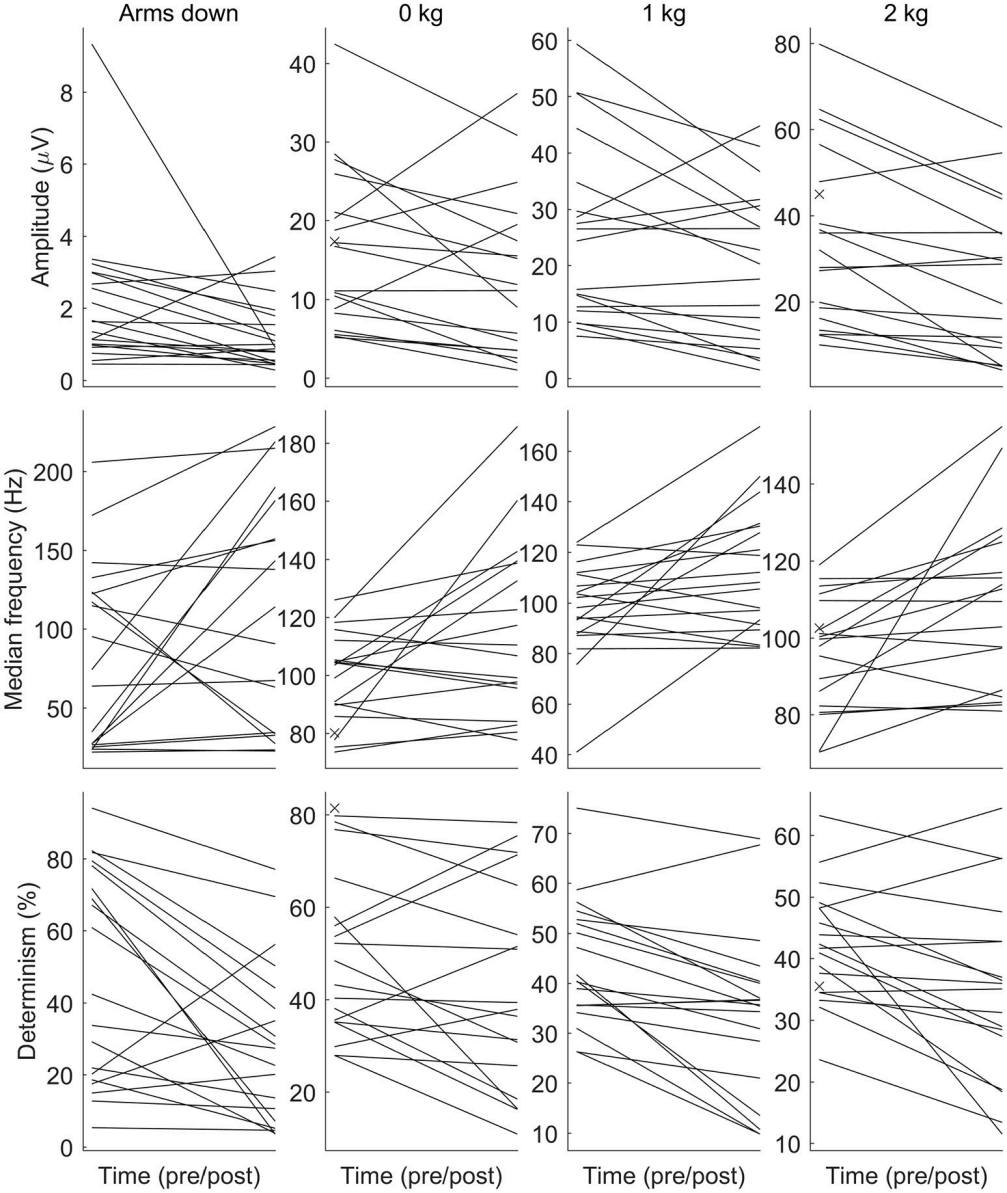
The amplitude (RMS), the median frequency, and the determinism of surface electromyogram before and after the dry immersion. The amplitude and the determinism tend to decrease, while the median frequency tends to increase after the dry immersion. Each patient is denoted by a line. Each subplot corresponds to a combination of a parameter (the row) and a loading (the column). Loading conditions are the following: arms down without any load (*Arms down*); *0/1/2 kg* loading on each hand with elbow being flexed to 90°. The muscle is the biceps of the left arm. The pre-immersion values are marked with crosses when the post-immersion counterpart is missing.

**Figure 4:**
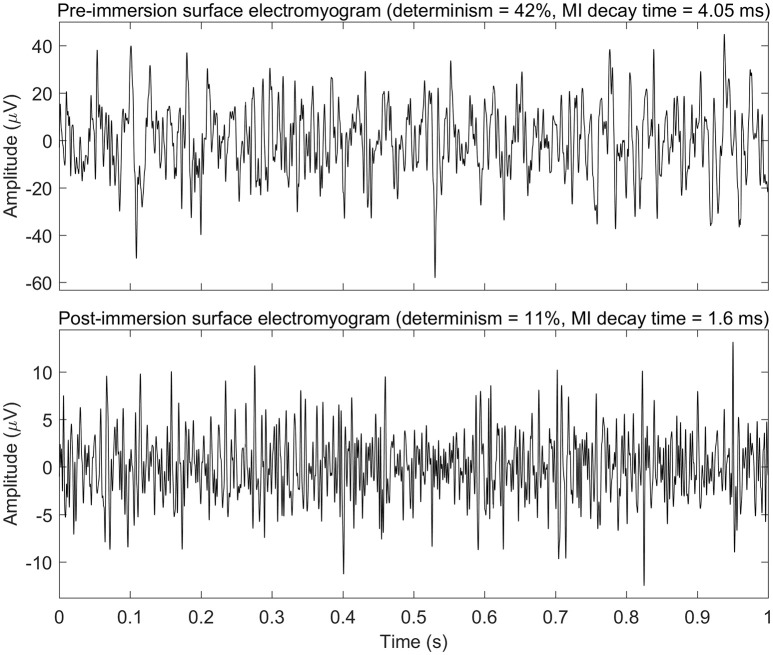
Example of surface electromyogram before and after the dry immersion procedure (patient No. 11 in Table 2; 1 kg loading; left arm). Only first second of the electromyograms is shown. This example demonstrates drop of amplitude, mutual information (MI) decay time, and determinism of electromyogram signal after the dry immersion. MI decay time may be linked to typical duration of turns of electromyogram, and determinism may be linked to regularity of electromyogram.

See Figure 2 for changes of the determinism for each patient. One can trace the tendency of determinism to decrease, which is more visible for the left biceps. For half of the patients (No. 2, 4, 8, 10, 11, 12, 14, 16, and 18), the determinism of recordings from the left biceps decreased no matter of the loading. What comes to the right biceps, the determinism values were often close to those for the left biceps, and the determinism changes were approximately parallel to those for the left biceps, although some exceptions occured, especially for the “arms down” loading. One may also consider that the changes near the bottom limit of determinism (patients No. 5, 11, 17, 19: “arms down” loading) are not very informative since determinism inherently cannot decrease below zero. See Figure 3 for general picture of changes of the amplitude, the median frequency, and the determinism. As for the determinism, the tendency of the amplitude to decrease and the tendency of the median frequency to increase were apparent for the left biceps only, so the pictures for the right biceps are not shown. See Figure 4 for an example of sEMG before and after the immersion. One can visually observe the drop of signal regularity and hence determinism.

The statistical analysis revealed that the following changes of the sEMG parameters were statistically significant. The amplitude decreased for both arms with the “arms down” loading and for the left arm with the 1 and 2 kg loadings. The median frequency increased for the left arm with the 1 and 2 kg loadings. Mutual information decay time decreased for the left arm with the 0 and 2 kg loadings. Sample entropy increased for both arms with the “arms down” loading and for the left arm with the 1 kg loading. Recurrence rate decreased for both arms with the “arms down” loading and for the left arm with the 1 kg loading. Determinism decreased for both arms with the “arms down” loading and for the left arm with the 1 and 2 kg loadings. The most statistically significant (*p* = 0.00084) change was for the determinism from the left biceps with 1 kg loading, which decreased for 16 out of 19 patients.

As regards the multiplicity of the statistical tests applied, the number of their results with *p* < 0.05 was enough to make an overall conclusion that sEMG parameters changed after the dry immersion. Indeed, if all the null hypotheses had been true then we would have had *p* < 0.05 for 5% of the hypotheses, but we had *p* < 0.05 for 32% of the hypotheses.

## 4. Discussion

The key hypothesis of this study postulated that a single one dry immersion (DI) exerts effect on the motor system in parkinsonian patients. Surface electromyogram (sEMG) signal during isometric contraction was assumed to be indicative of such effect. The major outcome of the present study was that the parameters of sEMG signal in parkinsonian patients indeed modified in the direction of improvement under a single DI session. Generally, we found that the amplitude and mutual information decay time of sEMG had decreased, while spectral frequencies had increased right after DI session. Among nonlinear dynamics based sEMG parameters, sample entropy had increased, while percent of determinism and recurrence rate had decreased after DI session. Correlation dimension proved insensitive to DI.

On one hand, the amplitude of sEMG is known to correlate with the number of active motor units and their firing rate (Farina et al., 2004). On the other hand, the rigidity in parkinsonian patients is often seen as “unwilled” muscle tension generated by uncontrolled firing of low-threshold (slow) motor units at rest (Cantello et al., 1995; Rossi et al., 1996). Therefore, we assume that lowered amplitude of sEMG in parkinsonian patients after DI session found in the present study may be attributed to decreased recruitment of these unconsciously active motor units, either at rest condition or under loading. Two neurophysiological mechanisms may have contributed to the decrease of sEMG amplitude after DI session.

First, sEMG amplitude may have decreased due to attenuation of the reflexive component of muscle tone. Such immersion-induced attenuation of the stretch reflex has been characteristic for another type of exaggerated muscle tone (spasticity) (Cronin et al., 2016). Similarly, in healthy subjects under a program of DI the reflex component of muscle tone has been diminished due to deprivation of excitatory tonic stimuli from the pacinian corpuscles of sole (Kozlovskaya et al., 1988; Navasiolava et al., 2011). In our recent study we have found that the rigidity subscore has indeed decreased under the program of short-term DI sessions (Meigal et al., 2018). However, the non-reflexive (mechano-elastic) component of muscle tone may also have contributed to the decrease of rigidity score. Further studying of the stretch reflex and viscoelasticity of muscles in PD patients under DI is needed to evaluate their contribution to hypotonic effect of DI.

Second, as muscle tone appears as muscle state of readiness to contraction (Davis et al., 2011) triggered by brainstem, basal ganglia, cerebellum, and cortical circuits in stressful conditions, we assume that central neural mechanisms might also have been involved in sEMG amplitude decreasing in PD patients during DI session. That is in good line with our earlier finding that after a program of DI the score of depression has significantly decreased (Meigal et al., 2018). As such, the DI condition has probably exerted its action on muscle hypertonicity (rigidity) via lowering the level of anxiety and thus relieving disinhibition of the stretch reflex. In an earlier study, Koryak (2002) have shown that electrically evoked maximal tetanic contraction force decreased by 8.2 after 7 days of DI, which suggests that most of the force loss was due to a reduction in motor drive. This supports the supraspinal application of DI action on sEMG parameters in PD patients. Similar decrease of central activation reflected in lower sEMG amplitude has been recently reported for the condition of hyperthermia (Coletta et al., 2018).

However, the amplitude of sEMG has decreased under DI at all loading conditions. That raises the question of whether sEMG amplitude in PD patients has decreased during DI due to inhibition of uncontrolled firing of “unwilled” motor units, or due to inhibition of voluntarily recruited motor units. The decrease of sEMG amplitude at loading was bigger in comparison with the decrease at the non-loading condition when arms were hanging down. Therefore, we assume that at loading conditions, besides these abnormally active “unwilled” motor units, some normally recruited motor units were also inhibited by DI. Passive heating has been already shown to inhibit spontaneous rest activity of motor units in PD patients (Meigal and Lupandin, 2005).

As for the nonlinear dynamics based sEMG parameters, several earlier studies have shown that in PD patients their values significantly differ from those in healthy controls. In particular, percent of recurrence rate and determinism of sEMG was higher in the PD group, while entropy and correlation dimension were lower in comparison to old and young healthy controls (Fattorini et al., 2005; Meigal et al., 2009). High percent of determinism reflects appearance of “recurrent fragments” on sEMG similar to one another, which is argued to originate from increased synchronization of motor units firing (Fattorini et al., 2005). In turn, such increased regularity of sEMG in PD indicates presence of mechanical tremor (Meigal et al., 2012). That is in line with growth of the spectral frequency of sEMG under certain loading conditions revealed in the present study, which may be associated with decreased synchronization of motor units (Fattorini et al., 2005). Therefore, shift of determinism and recurrence rate of sEMG after a single one DI session in the direction of lower values may be indicative of diminished synchronization of motor units firing and, hence, temporary inhibition of parkinsonian tremor. In our recent study we observed decrease of tremor score in some PD patients (Meigal et al., 2018). Still, direct accelerometric measurements are needed to validate that effect in a single DI session.

However, motor unit synchronization during muscle contraction is not a uniform phenomenon. Several forms of motor unit synchronization have been demonstrated (McAuley and Marsden, 2000). The first one is short-term synchronization (STS) that occurs within short time period (around 5 ms) as reflected with a narrow peak in the cross-correlogram (Kirkwood et al., 1982). The STS may either arise from the common axonal inputs to pairs of motoneurones (Sears and Stagg, 1976; Nordstrom et al., 1992) or be just an epiphenomenon of firing rate characteristics (Kline and de Luca, 2016). The other form of synchronization is long-term (or broad-peak) one (LTS) that occurs within broader time band (>20 ms) (Kirkwood et al., 1982; Freund, 1983). LTS comes into play when a large group of motoneurons starts to respond synchronically to a periodic synaptic input either of supraspinal or segmentary afferent origins (Kirkwood et al., 1982). Also, a tuned (or external) synchronization may arise when common inputs to a pair of motoneurones are themselves driven by a common input (McAuley and Marsden, 2000).

In PD, large part of motor units present specific firing pattern in a form of rhythmical groups of discharges (doublets and, occasionally, triplets). These groups are separated with longer interspike intervals and are associated with 4–6 Hz clustering on sEMG (Baker et al., 1992; Lupandin et al., 1993; Glendinning and Enoka, 1994). This pattern of firing is believed to largely contribute to generation of tremor in PD (Christakos et al., 2009). Doublets may also be associated with LTS that is seen as broad periodic correlations on cross-correlogram (Baker et al., 1992). As follows from these studies, both motor units synchrony and paired/tripled discharges may be responsible for greater values of determinism of sEMG in PD patients. STS has been shown to be greater in PD patients than in normal subjects but no evidence has been found that the higher incidence of STS in PD is a result of lower discharge rates of motor units or 4–6 Hz tremor that is the characteristic of PD patients (Baker et al., 1992).

Sample entropy has increased in some instances after the DI session. According to recent findings, sample entropy is higher when the motor units fire with higher variability of interspike intervals (Hwang et al., 2017).

The mutual information (MI) decay time, or first minimum of auto-mutual information function, quantifies how quickly information about a signal is lost as a function of time delay. MI decay time measures information transmission between a signal and its time shifted counterpart (Jeong et al., 2001) and, consequently, predictability of the signal (Ramanand et al., 2010). Also, MI decay time is regarded as a relevant estimator of irregularity of signal (Escudero et al., 2009). Though MI time decay has been already applied to measure information transmission within electroencephalographic signal (Jeong et al., 2001; Ramanand et al., 2010) and heart rate (Hoyer et al., 2005), to the best of our knowledge it has not yet been used to characterize sEMG. Correspondingly, neurophysiology behind MI decay time for sEMG has not yet been well understood. In our study, the MI decay time lasted from 1 to 13 ms, and it had tendency to decrease after DI session. Visual inspection of sEMG records showed that those with lower MI decay time values had shorter turns of sEMG curve, while those with higher values of MI decay time had longer turns (see Figure 4). Therefore, we assume that MI decay time may correlate with average duration of signal turns or longer patterns those are typical for a particular sEMG recording. Also, duration of MI decay time overlaps with that of STS. That prompts hypothetic association between these two parameters, because STS has been shown to be characteristic of PD patients (Baker et al., 1992). As such, we speculate that DI might decrease STS during DI session.

From several studies it is known that percent of determinism of sEMG responds to certain anti-PD therapies. For example, under dopaminergic anti-PD treatment percent of determinism of sEMG is decreased in comparison with the off-medication state (Rissanen et al., 2008; Ruonala et al., 2018). Similarly, after deep brain stimulation, sEMG signal characteristics turned more similar to the signal characteristics of the healthy controls (Rissanen et al., 2011). That is in good line with the result of the present study. We found only a few corresponding papers on the amount of motor unit synchrony after taking anti-PD medicine. In one, short-term synchrony was found to be irresponsible to taking levodopa (Baker et al., 1992). Therefore, we assume that analog microgravity induced by DI modulates the nonlinear dynamics based parameters of sEMG of parkinsonian patients by decreasing the long-term form of motor unit synchronization. We cannot exclude possible influence of DI also on the amount of motor unit doublets.

The most reliable neural site for effects of DI on motor unit synchrony and, hence, determinism of sEMG, presumably is located supraspinally, because (i) the above mentioned anti-PD therapies act directly on brain structures, and (ii) there is large corpus of data supporting the idea of supraspinal location of oscillatory circuits those generate the characteristic PD tremor (Timmermann et al., 2003). However, DI might also exerted certain peripheral action due to its well-documented unloading effect on muscles. We did not find corresponding data on nonlinear dynamics based parameters of sEMG during muscle contraction under the condition of real spaceflight. As for the physiological tremor, it is modified in the direction of lower frequency (as low as 4 Hz) and decreased amplitude by the 3rd day of space flight, presumably due to switch of main source of sensory information from load-dependent muscle reception to position-dependent joint reception (Gallasch et al., 1994). Thus, one may expect growth of regularity and decrease of amplitude of sEMG under the condition of real spaceflight. However, growth of determinism of sEMG was not the case in the present study. Several significant distinctions from real spaceflight condition could be figured out that probably contributed to such discrepancy. First, in our study, duration of analog microgravity was too short to induce the above said effect of microgravity on tremor. Second, arms under DI were not deprived of force-induced muscle afferent inputs. Additionally, in the present study, unlike to space based experiments, sEMG was collected under normal gravity, both before and after the session of DI. Thus, the condition of DI despite of its strong similarity with real microgravity cannot provide its absolute emulation.

The present study demonstrated large variance of sEMG parameters that might be associated with heterogeneity of clinical forms and severity of parkinsonism. Earlier, we demonstrated that percent of determinism of sEMG positively correlates with the UPDRS (part III) (Meigal et al., 2009) and tremor acceleration characteristic (Meigal et al., 2012). It means that with growing severity of PD tremor becomes more regular, while sEMG becomes more apparently clustered. We did not correlate percent of determinism of sEMG with the form of PD earlier, nor in this study due to insufficient number of parkinsonian patients with akinetic-rigid form. Further studies should have aimed on that.

### 4.1. Loading conditions

In the present study the most readily seen modifications of sEMG parameters were the characteristic of the condition when arms were freely hanging down without any load. Earlier we found that the most significant differences between PD and control groups were observed when no additional loading was placed on hands during the isometric elbow flexion task (Meigal et al., 2009). We concluded that additional loading applied on hands most likely revealed “regular” postural muscle tonus. In a way, under loading, sEMG of parkinsonian patients may have become more “normal” in comparison to the unloading condition. However, the results for the condition with arms freely hanging down should be treated with caution, because the sEMG amplitude was comparable to typical level of the noise generated on a fully relaxed biceps (Piervirgili et al., 2014).

### 4.2. Limitations

The major methodological limitation in the present study was small number of subjects who met all inclusion criteria (*n* = 19). Such relatively small number of parkinsonian patients and big number of factors (disease duration, clinical form, stage of disease, age, sex, medication) presumably affecting the outcome restricted opportunities of statistical analysis. Earlier experiments with younger subjects showed that the most effect of microgravity on muscle tone is observed by 2 h of staying of healthy subjects under the DI environment (Navasiolava et al., 2011). In this study, average age of patients was around 65 years. That restricts their tolerance to the DI procedure, mostly for the reason of urge to urinate and, in some instances, due to unstable blood pressure. Also, staying in DI reportedly provokes some compensated neurological signs (Navasiolava et al., 2011). In older subjects, especially neurologically ill, such hidden symptoms may have taken place. However, a 45 min long DI session was still enough to exert effect on sEMG parameters in parkinsonian patients.

In that study we omitted measuring UPDRS scores right after the DI session due to highly probable inference of cardiovascular factors, such as DI-induced decrease of blood pressure and orthostatic reactions, on motor signs (gait, postural reactions, muscle tone) in PD patients. From our earlier study we know that muscle rigidity subtotal of UPDRS-III score and, to lesser degree, tremor subtotal, decrease across the program of DI (Meigal et al., 2018). In further studies, it would be wise to correlate sEMG parameters with clinical symptoms and scales that characterize parkinsonian patients for better understanding of the association between sEMG and motor symptoms. The idiopathic Parkinson's disease and vascular parkinsonism must be compared in their compliance to DI therapy. Additionally, it would be of interest to trace the net effect of several, rather than one, DI sessions on sEMG.

### 4.3. In conclusion

The results presented here provide promising evidence that such analog microgravity environment as dry immersion exerts significant influence on the motor system in parkinsonian patients, as it is seen from surface electromyogram modification after a single short-term dry immersion session. The major outcome of the study was that the amplitude, mutual information decay time, recurrence rate, and determinism of surface electromyogram decreased whereas median frequency and sample entropy, in contrast, increased right after dry immersion. This evidences decreased rigidity and weakened tremor seen as synchronicity of motor unit activity.

## Author contributions

GM has contributed by data analysis and interpretation, and by writing of the manuscript. AM has contributed by the research conception, design of the work, data acquisition and interpretation, and by writing of the manuscript. IS and LC have contributed by the research conception, design of the work, and by critical revision of the manuscript. LG-M has contributed by design of the work, data acquisition, and by writing of the manuscript. NS has contributed by design of the work, data acquisition, and by critical revision of the manuscript. SR and PK have contributed by data analysis and interpretation, and by critical revision of the manuscript. All the authors have read and approved the manuscript before the publication and agree to be accountable for all aspects of the research.

### Conflict of interest statement

The authors declare that the research was conducted in the absence of any commercial or financial relationships that could be construed as a potential conflict of interest.

## References

[B1] Alves G. Wentzel-Larsen T. Aarsland D. Larsen J. P. (2005). Progression of motor impairment and disability in Parkinson disease: a population-based study. Neurology 65, 1436–1441. 10.1212/01.wnl.0000183359.50822.f2 16275832

[B2] Baker J. R. Davey N. J. Ellaway P. H. Friediland C. L. (1992). Short-term synchrony of motor unit discharge during weak isometric contraction in Parkinson's disease. Brain 115, 137–154. 10.1093/brain/115.1.137 1559149

[B3] Baradaran N. Tan S. N. Liu A. Ashoori A. Palmer S. J. Wang Z. J.. (2013). Parkinson's disease rigidity: relation to brain connectivity and motor performance. Front. Neurol. 4:67. 10.3389/fneur.2013.00067 23761780 PMC3672800

[B4] Boccia G. Dardanello D. Beretta-Piccoli M. Cescon C. Coratella G. Rinaldo N. Barbero M.. (2016). Muscle fiber conduction velocity and fractal dimension of EMG during fatiguing contraction of young and elderly active men. Physiol. Meas. 37, 162–174. 10.1088/0967-3334/37/1/162 26684024

[B5] Boersma I. Jones J. Carter J. Bekelman D. Miyasaki J. Kutner J.. (2016). Parkinson disease patients' perspectives on palliative care needs: what are they telling us? Neurol. Clin. Pract. 6, 209–219. 10.1212/CPJ.0000000000000233 27347438 PMC4909525

[B6] Buehring B. Belavý D. L. Michaelis I. Gast U. Felsenberg D. Rittweger J. (2011). Changes in lower extremity muscle function after 56 days of bed rest. J. Appl. Physiol. 111, 87–94. 10.1152/japplphysiol.01294.2010 21527664

[B7] Bukowska A. A. Kreżałek P. Mirek E. Bujas P. Marchewka A. (2016). Neurologic music therapy training for mobility and stability rehabilitation with Parkinson's disease – a pilot study. Front. Hum. Neurosci. 9:710. 10.3389/fnhum.2015.00710 26858628 PMC4726780

[B8] Cantello R. Gianelli M. Civardi C. Mutani R. (1995). Parkinson's disease rigidity: EMG in a small hand muscle at “rest”. Electroencephalogr. Clin. Neurophysiol. 97, 215–222. 10.1016/0013-4694(95)93574-Q 7489682

[B9] Celucci C. J. Albano A. M. Rapp P. E. (2003). Comparative study of embedding methods. Phys. Rev. E 67:066210 10.1103/PhysRevE.67.066210 16241329

[B10] Cescon C. Gazzoni M. (2010). Short term bed-rest reduces conduction velocity of individual motor units in leg muscles. J. Electromyogr. Kines. 20, 860–867. 10.1016/j.jelekin.2010.03.008 20403710

[B11] Christakos C. N. Erimaki S. Anagnostou E. Anastasopoulos D. (2009). Tremor-related motor unit firing in Parkinson's disease: implications for tremor genesis. J. Physiol. 587, 4811–4827. 10.1113/jphysiol.2009.173989 19703960 PMC2770149

[B12] Coletta N. A. Mallette M. M. Gabriel D. A. Tyler C. J. Cheung S. S. (2018). Core and skin temperature influences on the surface electromyographic responses to an isometric force and position task. PLoS ONE 13:e0195219. 10.1371/journal.pone.0195219 29596491 PMC5875857

[B13] Cronin N. J. Valtonen A. M. Waller B. Pöyhönen T. Avela J. (2016). Effects of short term water immersion on peripheral reflex excitability in hemiplegic and healthy individuals: a preliminary study. J. Musculoskel. Neuron. 16, 58–62. Available online at: http://www.ismni.org/jmni/pdf/63/V16I1_09CRONIN.pdf 26944824 PMC5089456

[B14] Davis J. R. Horslen B. C. Nishikawa K. Fukushima K. Chua R. Inglis J. T.. (2011). Human proprioceptive adaptations during states of height-induced fear and anxiety. J. Neurophysiol. 106, 3082–3090. 10.1152/jn.01030.2010 21918000

[B15] de Dreu M. J. Kwakkel G. van Wegen E. E. H. (2015). Partnered dancing to improve mobility for people with Parkinson's disease. Front. Neurosci. 9:444. 10.3389/fnins.2015.00444 26696808 PMC4675848

[B16] de Lau L. M. L. Breteler M. M. B. (2006). Epidemiology of Parkinson's disease. Lancet Neurol. 5, 525–535. 10.1016/S1474-4422(06)70471-9 16713924

[B17] Demangel R. Treffel L. Py G. Brioche T. Pagano A. F. Bareille M.-P.. (2017). Early structural and functional signature of 3-day human skeletal muscle disuse using the dry immersion model. J. Physiol. 595, 4301–4315. 10.1113/JP273895 28326563 PMC5491890

[B18] di Biase L. Summa S. Tosi J. Taffoni F. Marano M. Rizzo A. C.. (2018). Quantitative analysis of bradykinesia and rigidity in Parkinson's disease. Front. Neurol. 9:121. 10.3389/fneur.2018.00121 29568281 PMC5853013

[B19] Dietz V. Sinkjaer T. (2007). Spastic movement disorder: impaired reflex function and altered muscle mechanic. Lancet Neurol. 6, 725–733. 10.1016/S1474-4422(07)70193-X 17638613

[B20] Ebersbach G. Edler D. Kaufhold O. Wissel J. (2008). Whole body vibration versus conventional physiotherapy to improve balance and gait in Parkinson's disease. Arch. Phys. Med. Rehabil. 89, 399–403. 10.1016/j.apmr.2007.09.031 18295614

[B21] Escudero J. Hornero R. Absolo D. (2009). Interpretation of the auto-mutual information rate of decrease in the context of biomedical signal analysis. Application to electroencephalogram recordings. Physiol. Meas. 30, 187–199. 10.1088/0967-3334/30/2/006 19147896

[B22] Fan J.-Y. Chang B.-L. Wu Y.-R. (2016). Relationships among depression, anxiety, sleep, and quality of life in patients with Parkinson's disease in Taiwan. Parkinson's Dis. 2016:4040185. 10.1155/2016/4040185 27293956 PMC4884599

[B23] Farina D. Merletti R. Enoka R. M. (2004). The extraction of neural strategies from the surface EMG. J. Appl. Physiol. 96, 1486–1495. 10.1152/japplphysiol.01070.2003 15016793

[B24] Farina D. Merletti R. Enoka R. M. (2014). The extraction of neural strategies from the surface EMG: an update. J. Appl. Physiol. 117, 1215–1230. 10.1152/japplphysiol.00162.2014 25277737 PMC4254845

[B25] Fattorini L. Felici F. Filligoi G. C. Traballesi M. Farina D. (2005). Influence of high motor unit synchronization levels on non-linear and spectral variables of the surface EMG. J. Neurosci. Methods 143, 133–139. 10.1016/j.jneumeth.2004.09.018 15814145

[B26] Fraser A. M. Swinney H. L. (1986). Independent coordinates for strange attractors from mutual information. Phys. Rev. A 33, 1134–1140. 10.1103/PhysRevA.33.1134 9896728

[B27] Freund H. J. (1983). Motor unit and muscle activity in voluntary motor control. Physiol. Rev. 63, 387–436. 10.1152/physrev.1983.63.2.387 6340133

[B28] Fu A. Wang C. Qi H. Li F. Wang Z. He F. (2016). Electromyography-based analysis of human upper limbs during 45-day head-down bed-rest. Acta Astronaut. 120, 260–269. 10.1016/j.actaastro.2015.12.007

[B29] Gallasch E. Kozlovskaya I. Löscher W. N. Konev A. Kenner T. (1994). Arm tremor and precision of hand force control in a short and long term flight on the Mir-Space-Station. Acta Astronaut. 33, 49–55. 10.1016/0094-5765(94)90108-2 11539538

[B30] Gao J. Zheng Z. (1994). Direct dynamical test for deterministic chaos and optimal embedding of a chaotic time series. Phys. Rev. E 49, 3807–3814. 10.1103/PhysRevE.49.3807 9961667

[B31] Glendinning D. S. Enoka R. M. (1994). Motor unit behavior in Parkinson's disease. Phys. Ther. 74, 61–70. 10.1093/ptj/74.1.61 8265729

[B32] Gordon J. (1990). Disorders of motor control, in Key Issues in Neurological Physiotherapy, eds Ada L. Canning C. (London: Heinemann), 35.

[B33] Grassberger P. Procaccia I. (1983). Characterization of Strange Attractors. Phys. Rev. Lett. 50, 346–349. 10.1103/PhysRevLett.50.346

[B34] Guyton A. C. Hall J. E. (2011). Textbook of Medical Physiology, 12th Edn Philadelphia, PA: Saunders Elsevier.

[B35] Hoyer D. Pompe B. Chon K. H. Hardraht H. Wicher C. Zwiener U. (2005). Mutual information function assesses autonomic information flow of heart rate dynamics at different time scales. IEEE Trans. Biomed. Eng. 52, 584–592. 10.1109/TBME.2005.844023 15825860

[B36] Hwang I.-S. Lin Y.-T. Huang W.-M. Yang Z.-R. Hu C.-L. Chen Y.-C. (2017). Alterations in neural control of constant isometric contraction with the size of error feedback. PLoS ONE 12:e0170824. 10.1371/journal.pone.0170824 28125658 PMC5268650

[B37] Jeong J. Gore J. C. Peterson B. S. (2001). Mutual information analysis of the EEG in patients with Alzheimer's disease. Clin. Neurophysiol. 112, 827–835. 10.1016/S1388-2457(01)00513-2 11336898

[B38] Katsura Y. Yoshikawa T. Ueda S.-Y. Usui T. Sotobayashi D. Nakao H.. (2010). Effects of aquatic exercise training using water-resistance equipment in elderly. Eur. J. Appl. Physiol. 108, 957–964. 10.1007/s00421-009-1306-0 19960351

[B39] Katz R. T. Rymer W. Z. (1989). Spastic hypertonia: mechanisms and measurement. Arch. Phys. Med. Rehabil. 70, 144–155. 2644919

[B40] Kennel M. B. Brown R. Abarbanel H. D. I. (1992). Determining embedding dimension for phase-space reconstruction using a geometrical construction. Phys. Rev. A 45, 3403–3411. 10.1103/PhysRevA.45.3403 9907388

[B41] Kirkwood P. A. Sears T. A. Tuck D. L. Westgaard R. H. (1982). Variations in the time course of the synchronization of intercostal motoneurones in the cat. J. Physiol. 327, 105–135. 10.1113/jphysiol.1982.sp014223 7120134 PMC1225100

[B42] Kline J. C. de Luca C. J. (2016). Synchronization of motor unit firings: an epiphenomenon of firing rate characteristics not common inputs. J. Neurophysiol. 115, 178–192. 10.1152/jn.00452.2015 26490288 PMC4760486

[B43] Koryak Y. A. (2002). Surface action potential and contractile properties of the human triceps surae muscle: effect of 'dry' water immersion. Exp. Physiol. 87, 101–111. 10.1113/eph8702301 11805864

[B44] Kozlovskaya I. Dmitrieva I. Grigorieva L. Kirenskaya A. Kreidich Yu. (1988). Gravitational mechanisms in the motor system. Studies in real and simulated weightlessness, in Stance and Motion: Facts and Concepts, eds Gurfinkel V. S. Ioffe M. E. Massion J. Roll J. P. (Boston, MA: Springer), 37–48.

[B45] Lupandin Yu. V. Antonen E. G. Meigal Yu. A. (1993). The patterns of bioelectrical activity of motor units in different forms of parkinsonism. Zh. Nevrol. Psikhiatr. Im. S. S. Korsakova 93, 30–34. 8160498

[B46] Mañé R. (1981). On the dimension of the compact invariant sets of certain nonlinear maps, in Dynamical Systems and Turbulence, Lecture Notes in Mathematics, vol. 898, eds Rand D. A. Young L.-S. (Warwick: Springer-Verlag), 230–242.

[B47] Marwan N. Romano M. C. Thiel M. Kurths J. (2007). Recurrence plots for the analysis of complex systems. Phys. Rep. 438, 237–329. 10.1016/j.physrep.2006.11.001

[B48] McAuley J. H. Marsden C. D. (2000). Physiological and pathological tremors and rhythmic central motor control. Brain 123, 1545–1567. 10.1093/brain/123.8.1545 10908186

[B49] Meigal A. Gerasimova-Meigal L. Saenko I. Subbotina N. (2018). Dry immersion as a novel physical therapeutic intervention for rehabilitation of Parkinson's disease patients: a feasibility study. Phys. Med. Rehab. Kuror. 10.1055/a-0577-5139

[B50] Meigal A. Lupandin Y. (2005). "Thermoregulation-dependent component" in pathophysiology of motor disorders in Parkinson's disease? Pathophysiology 11, 187–196. 10.1016/j.pathophys.2005.02.001 15837163

[B51] Meigal A. Miroshnichenko G. Kuzmina A. Rissanen S. Georgiadis S. Karjalainen P. (2015). Nonlinear parameters of surface EMG in schizophrenia patients depend on kind of antipsychotic therapy. Front. Physiol. 6:197. 10.3389/fphys.2015.00197 26217236 PMC4498039

[B52] Meigal A. Yu. Rissanen S. Tarvainen M. Georgiadis S. D. Karjalainen P. A. Airaksinen O.. (2012). Linear and nonlinear tremor acceleration characteristics in patients with Parkinson's disease. Physiol. Meas. 33, 395–412. 10.1088/0967-3334/33/3/395 22370008

[B53] Meigal A. Yu. Rissanen S. Tarvainen M. Kajalainen P. A. Iudina-Vassel I. A. Airaksinen O.. (2009). Novel parameters of surface EMG in patients with Parkinson's disease and healthy young and old controls. J. Electromyogr. Kines. 19, e206–e213. 10.1016/j.jelekin.2008.02.008 18407522

[B54] Meigal A. Yu. Rissanen S. M. Tarvainen M. P. Airaksinen O. Kankaanpää M. Karjalainen P. A. (2013). Non-linear EMG parameters for differential and early diagnostics of Parkinson's disease. Front. Neurol. 4:135. 10.3389/fneur.2013.00135 24062722 PMC3775312

[B55] Mewett D. T. Reynolds K. J. Nazeran H. (2004). Reducing power line interference in digitised electromyogram recordings by spectrum interpolation. Med. Biol. Eng. Comput. 42, 524–531. 10.1007/BF02350994 15320462

[B56] Miyai I. Fujimoto Y. Ueda Y. Yamamoto H. Nozaki S. Saito T.. (2000). Treadmill training with body weight support: its effect on Parkinson's disease. Arch. Phys. Med. Rehabil. 81, 849–852. 10.1053/apmr.2000.4439 10895994

[B57] Mulder E. R. Gerrits K. H. L. Kleine B. U. Rittweger J. Felsenberg D. de Haan A. (2009). High-density surface EMG study on the time course of central nervous and peripheral neuromuscular changes during 8 weeks of bed rest with or without resistive vibration exercise. J. Electromyogr. Kines. 19, 208–218. 10.1016/j.jelekin.2007.04.002 17560125

[B58] Navasiolava N. M. Custaud M.-A. Tomilovskaya E. S. Larina I. M. Mano T. Gauquelin-Koch G.. (2011). Long-term dry immersion: review and prospects. Eur. J. Appl. Physiol. 111, 1235–1260. 10.1007/s00421-010-1750-x 21161267

[B59] Needle A. R. Baumeister J. Kaminski T. W. Higginson J. S. Farquhar W. B. Swanik C. B. (2014). Neuromechanical coupling in the regulation of muscle tone and joint stiffness. Scand. J. Med. Sci. Sports 24, 737–748. 10.1111/sms.12181 25371932

[B60] Ni M. Signorile J. F. Mooney K. Balachandran A. Potiaumpai M. Luca C.. (2016). Comparative effect of power training and high-speed yoga on motor function in older patients with Parkinson disease. Arch. Phys. Med. Rehabil. 97, 345–354.e15. 10.1016/j.apmr.2015.10.095 26546987

[B61] Nordstrom M. A. Fuglevand A. J. Enoka R. M. (1992). Estimating the strength of common input to human motoneurons from the cross-correlogram. J. Physiol. 453, 547–574. 10.1113/jphysiol.1992.sp019244 1464844 PMC1175573

[B62] Noyes K. Liu H. Li Y. Holloway R. Dick A. W. (2006). Economic burden associated with Parkinson's disease on elderly Medicare beneficiaries. Mov. Disord. 21, 362–372. 10.1002/mds.20727 16211621

[B63] Oung Q. W. Muthusamy H. Lee H. L. Basah S. N. Yaacob S. Sarillee M.. (2015). Technologies for assessment of motor disorders in Parkinson's disease: a review. Sensors 15, 21710–21745. 10.3390/s150921710 26404288 PMC4610449

[B64] Picelli A. Tamburin S. Passuello M. Waldner A. Smania N. (2014). Robot-assisted arm training in patients with Parkinson's disease: a pilot study. J. NeuroEng. Rehabil. 11, 1–4. 10.1186/1743-0003-11-28 24597524 PMC3973978

[B65] Piervirgili G. Petracca F. Merletti R. (2014). A new method to assess skin treatments for lowering the impedance and noise of individual gelled Ag-AgCl electrodes. Physiol. Meas. 35, 2101–2118. 10.1088/0967-3334/35/10/2101 25243492

[B66] Portero P. Vanhoutte C. Goubel F. (1996). Surface electromyogram power spectrum changes in human leg muscles following 4 weeks of simulated microgravity. Eur. J. Appl. Physiol. O. 73, 340–345. 10.1007/BF02425496 8781866

[B67] Ramanand P. Bruce M. C. Bruce E. N. (2010). Mutual information analysis of EEG signals indicates age-related changes in cortical interdependence during sleep in middle-aged vs. elderly women. J. Clin. Neurophysiol. 27, 274–284. 10.1097/WNP.0b013e3181eaa9f5 20634711 PMC3037822

[B68] Richman J. S. Moorman J. R. (2000). Physiological time-series analysis using approximate entropy and sample entropy. Am. J. Physiol. Heart Circ. Physiol. 278, H2039–H2049. 10.1152/ajpheart.2000.278.6.H2039 10843903

[B69] Riley M. A. van Orden G. C. editors (2005). Tutorials in Contemporary Nonlinear Methods for the Behavioral Science. Available online at: https://www.nsf.gov/pubs/2005/nsf05057/nmbs/nmbs.pdf (Accessed June 21, 2018).

[B70] Rissanen S. M. Kankaanpää M. Meigal A. Tarvainen M. P. Nuutinen J. Tarkka I. M.. (2008). Surface EMG and acceleration signals in Parkinson's disease: feature extraction and cluster analysis. Med. Biol. Eng. Comput. 46, 849–858. 10.1007/s11517-008-0369-0 18633662

[B71] Rissanen S. M. Kankaanpää M. Tarvainen M. P. Novak V. Novak P. Hu K.. (2011). Analysis of EMG and acceleration signals for quantifying the effects of deep brain stimulation in Parkinson's disease. IEEE Trans. Biomed. Eng. 58, 2545–2553. 10.1109/TBME.2011.2159380 21672674 PMC3873135

[B72] Rivelis Y. Morice K. (2018). Spasticity, in StatPearls [Internet]. Treasure Island: StatPearls Publishing Available online at: https://www.ncbi.nlm.nih.gov/books/NBK507869/ (Updated June 19, 2018).

[B73] Rossi B. Siciliano G. Carboncini M. C. Manca M. L. Massetani R. Viacava P.. (1996). Muscle modifications in Parkinson's disease: myoelectric manifestations. Electroencephalogr. Clin. Neurophysiol. 101, 211–218. 10.1016/0924-980X(96)94672-X 8647033

[B74] Ruonala V. Pekkonen E. Airaksinen O. Kankaanpää M. Karjalainen P. A. Rissanen S. M. (2018). Levodopa-induced changes in electromyographic patterns in patients with advanced Parkinson's disease. Front. Neurol. 9:35. 10.3389/fneur.2018.00035 29459845 PMC5807331

[B75] Sáchez-Ferro Á. Elshehabi M. Godinho C. Salkovic D. Hobert M. A. Domingos J. (2016). New methods for the assessment of Parkinson's disease (2005 to 2015): a systematic review. Movement Disord. 31, 1283–1292. 10.1002/mds.26723 27430969

[B76] Scandalis T. A. Bosak A. Berliner J. C. Helman L. L. Wells M. R. (2001). Resistance training and gait function in patients with Parkinson's disease. Am. J. Phys. Med. Rehabil. 80, 38–43. 10.1097/00002060-200101000-00011 11138953

[B77] Schneider S. Peipsi A. Stokes M. Knicker A. Abeln V. (2015). Feasibility of monitoring muscle health in microgravity environments using Myoton technology. Med. Biol. Eng. Comput. 53, 57–66. 10.1007/s11517-014-1211-5 25331739

[B78] Sears T. A. Stagg D. (1976). Short-term synchronization of intercostal motoneurone activity. J. Physiol. 263, 357–381. 10.1113/jphysiol.1976.sp011635 1018273 PMC1307707

[B79] Sturman M. M. Vaillancourt D. E. Corcos D. M. (2005). Effects of aging on the regularity of physiological tremor. J. Neurophysiol. 93, 3064–3074. 10.1152/jn.01218.2004 15716367

[B80] Sung P. S. Zurcher U. Kaufman M. (2008). Gender differences in spectral and entropic measures of erector spinae muscle fatigue. J. Rehabil. Res. Dev. 45, 1431–1440. 10.1682/JRRD.2007.11.0196 19319765

[B81] Takens F. (1981). Detecting strange attractors in turbulence, in Dynamical Systems and Turbulence, Lecture Notes in Mathematics, vol. 898, eds Rand D. A. Young L.-S. (Berlin; Heidelberg: Springer-Verlag), 366–381.

[B82] Tarvainen M. P. Ranta-aho P. O. Karjalainen P. A. (2002). An advanced detrending method with application to HRV analysis. IEEE Trans. Biomed. Eng. 49, 172–175. 10.1109/10.979357 12066885

[B83] The Parkinson Study Group (2004). Levodopa and the progression of Parkinson's disease. N. Engl. J. Med. 351, 2498–2508. 10.1056/NEJMoa033447 15590952

[B84] Timmermann L. Gross J. Dirks M. Volkmann J. Freund H.-J. Schnitzler A. (2003). The cerebral oscillatory network of parkinsonian resting tremor. Brain 126, 199–212. 10.1093/brain/awg022 12477707

[B85] Tomlinson C. L. Herd C. P. Clarke C. E. Meek C. Patel S. Stowe R. (2014). Physiotherapy for Parkinson's disease: a comparison of techniques. Cochrane Db. Syst. Rev. 6:CD002815 10.1002/14651858.CD002815.pub2 PMC7120367 24936965

[B86] Trompetto C. Marinelli L. Mori L. Pelosin E. Currà A. Molfetta L.. (2014). Pathophysiology of spasticity: implications for neurorehabilitation. Biomed. Res. Int. 2014:354906. 10.1155/2014/354906 25530960 PMC4229996

[B87] Vaillancourt D. E. Prodoehl J. Metman L. V. Bakay R. A. Corcos D. M. (2004). Effects of deep brain stimulation and medication on bradykinesia and muscle activation in Parkinson's disease. Brain 127, 491–504. 10.1093/brain/awh057 14662520

[B88] Vivas J. Arias P. Cudeiro J. (2011). Aquatic therapy versus conventional land-based therapy for Parkinson's disease: an open-label pilot study. Arch. Phys. Med. Rehabil. 92, 1202–1210. 10.1016/j.apmr.2011.03.017 21807139

[B89] Volpe D. Giantin M. G. Maestri R. Frazzitta G. (2014). Comparing the effects of hydrotherapy and land-based therapy on balance in patients with Parkinson's disease: a randomized controlled pilot study. Clin. Rehabil. 28, 1210–1217. 10.1177/0269215514536060 24895382

[B90] Ward A. B. (2000). Assessment of muscle tone. Age Ageing 29, 385–386. 10.1093/ageing/29.5.385 11108407

[B91] Watenpaugh D. E. (2016). Analogs of microgravity: head-down tilt and water immersion. J. Appl. Physiol. 120, 904–914. 10.1152/japplphysiol.00986.2015 26869710

[B92] Yen C. Y. Lin K. H. Hu M. H. Wu R. M. Lu T. W. Lin C. H. (2011). Effects of virtual reality-augmented balance training on sensory organization and attentional demand for postural control in people with Parkinson disease: a randomized controlled trial. Phys. Ther. 91, 862–874. 10.2522/ptj.20100050 21474638

[B93] Zhou J. Yin T. Gao Q. Yang X. C. (2015). A meta-analysis on the efficacy of tai chi in patients with Parkinson's disease between 2008 and 2014. Evid. Based Compl. Alt. 2015:593263. 10.1155/2015/593263 25649281 PMC4306407

[B94] Zhu H. Lu Z. Jin Y. Duan X. Teng J. Duan D. (2015). Low-frequency repetitive transcranial magnetic stimulation on Parkinson motor function: a meta-analysis of randomised controlled trials. Acta Neuropsychiatr. 27, 82–89. 10.1017/neu.2014.43 25592544

